# Policy options and practical recommendations for determining priorities in public health research agendas in peripheral countries: insights from a collaborative work initiative in Argentina during the COVID-19 pandemic

**DOI:** 10.3389/fmed.2023.1334194

**Published:** 2024-01-11

**Authors:** Natalia Brenda Fernández, María Georgina Herrera, Matías Blaustein, María Florencia Pignataro

**Affiliations:** ^1^Instituto de Biociencias, Biotecnología y Biología traslacional (iB3), Departamento de Fisiología, Biología Molecular y Celular (DFBMC), Facultad de Ciencias Exactas y Naturales (FCEyN), Universidad de Buenos Aires (UBA), Buenos Aires, Argentina; ^2^Consejo Nacional de Investigaciones Científicas y Técnicas (CONICET), Buenos Aires, Argentina

**Keywords:** COVID-19 pandemic, Argentina, scientific funding, public policy issues, collaborative networks

## 1 Introduction

The COVID-19 pandemic produced by the newly emerged coronavirus SARS-CoV-2 changed public health agendas and scientific priorities (1). During most of 2020, no vaccines or therapies were available to fight the acute respiratory disease produced by this new type of coronavirus (2). This uncertain situation led scientists to increase interdisciplinary collaborations in order to contribute to the understanding of SARS-CoV-2 infection. Therefore, several new biotechnological initiatives were carried out in extraordinary time to generate tools that could help in prevention, diagnosis and therapeutics (3). The majority of them were developed in central countries and resulted in several approaches that were distributed worldwide. However, peripheral countries, like Argentina, Brazil, Cuba, and India, have also made their own developments providing resources to local production necessary to fight against this respiratory disease (4, 5).^1^,^2^ One of these initiatives was the Argentinean AntiCovid Consortium, where we partnered with nearly 30 researchers (PIs, young researchers, postdocs and PhD students) from different scientific backgrounds, combining our knowledge and expertise to carry out a multidisciplinary strategy.^3^ The main objective of this Consortium was to rapidly generate scalable and economically accessible biotechnological tools. In particular, we focused on the receptor binding domain (RBD) of the SARS-CoV-2 Spike protein, which was employed for local development of *in vitro* diagnostic kits and later as an antigen for vaccine development. One characteristic of the consortium was to work as horizontally as possible (each one according to his/her possibilities during the pandemic), without establishing hierarchies among members beyond those given by experience and knowledge. In line with this vision, some of the biotechnological outcomes of the consortium were published in open access peer-reviewed journals, listing the authors in alphabetical order along with an equal contribution statement (6, 7), to make the developments available to the scientific community and the society in general. In this article we will comment on the positive outcomes of this initiative, some of the drawbacks we encountered, as well as open questions and perspectives on the role of science in peripheral countries.

## 2 Results

### 2.1 Positive outcomes of the AntiCovid Consortium collaborative initiative

During the pandemic lockdown in Argentina, most research laboratories from universities were only open to work on COVID-19 related topics (8).^4^ Particularly, the members of this collaborative initiative reorganized ongoing research projects to work on RBD protein from SARS-CoV-2 production and characterization. This research was initially funded by personal donations channeled by the Exact and Natural Sciences Foundation^5^ and a few months later the project was partially financed by a special call for COVID-19 projects from the Argentinian National Agency for the Promotion of Research, Technological Development and Innovation.^6^

The main goal of this initiative was to produce and characterize SARS-CoV-2 antigens in different and economically accessible biological systems (human cell lines, plants, amoeba, yeast and bacteria) to meet national and regional needs. In this regard, the yeast *Pichia Pastoris* fulfilled most of the requirements as an antigen expression system. In a 6 months period, the first collaborative article condensing part of the work on the characterization of the RBD of SARS-CoV-2 produced by HEK-293T human cells and in *Pichia Pastoris* was sent for publication (6). During this period, we also supplied high quality RBD antigen (through University MTAs) to 9 different institutions across Argentina for use in their basic and applied research projects (9, 10).^7^ In the meantime, members of the Consortium took part in several outreach activities where not only general COVID-19 information was shared but also results and work perspectives (11).^8^,^9^ Importantly, some results generated by the AntiCovid Consortium contributed to one of the four COVID-19 vaccine project candidates (project called ARGENVAC) funded by the Argentinian Ministry of Science and Technology (MinCyT) to progress in preclinical studies.^10^ Moreover, the RBD protein produced by this initiative was the main antigen of an *in vitro* diagnostic kit developed in collaboration with other Public Institutions and one private partner.^11^

As an additional outcome, researchers established a previously unimplemented network from different bio-scientific fields, fostering new collaborative and positive interactions among their peers. After a fruitful development, reaching the objectives of the project, in March 2021, the Consortium members decided to conclude this initiative. Nevertheless, some researchers continued working on related outcomes of the RBD production and usage as antigen, while others resumed their prior lines of research.

### 2.2 Difficulties in the implementation of an extended scientific collaborative initiative in a peripheral country, such as Argentina

At the onset of the pandemic, the local production of RBD required the exploration of different biological systems to select one capable of producing this domain fragment with the highest quality, and in a scalable manner for local implementation. The Consortium initiative received financial assistance from private donors and from the local scientific public system. However, in the latter case, the support was delayed, and the budget was less than expected, leaving personal contributions crucial during the initial stages.

We also encountered many challenges during the technology transfer process. Firstly, apart from the willingness to collaborate between all parties, the articulation between different actors of the public systems [Public Universities, National Research Council (CONICET), Public Hospitals, National Laboratories Network, etc.] found several bureaucracies related setbacks that also delayed the transferences. Secondly, even though efforts were pursued, at first, to promote technology transfer between public sectors (i.e., toward Public Laboratories, nucleated by the National Agency of Public Laboratories), the transference to the private sector was far more promoted at every stage of the process. This is partially because the pre-established capacities needed to rapidly adopt the transference were not always met in the Public sector. However, the path to find private national partners was also difficult given the Argentinian economic situation and instability. Moreover, the limited connections between universities and industry and the bureaucratic hurdles associated with establishing those interactions with productive/industrial sectors did not help, holding back the developments. Importantly, intellectual property barriers should be taken into account when addressing the sanitary, social and economic outcomes of collaborative research.

On another aspect, a significant part of the local scientific community itself was unenthusiastic and had reservations toward this type of horizontal, multidisciplinary collaborative work. The alphabetical order listing and “equal authorship contribution” stated in the submitted articles by the consortium was received with skepticism during the peer review process when applying for permanent positions or promotions. In some cases, it was even considered a minor contribution, arguing that it was not part of the applicant's specific field/subject of research, while analytical, bioinformatics or theoretical work done at home by other researchers who continued working on their research lines, and did not perform research on COVID-19, was considered a major contribution. This is a disincentive, in practical terms, to work on topics of public interest for future health emergencies.

### 2.3 Practical recommendations to establish priorities and connections between the scientific and health research agendas, especially during sanitary emergencies

The COVID-19 pandemic left several new opportunities for the development and improvement of biotechnological tools in peripheral countries. Particularly, regarding vaccine development, several countries, such as India and Brazil, have shown to be able not only to produce at large scales those developed in central countries and transferred through licenses (12),^12^ but also to promote local research in the field, producing their own vaccines, as in the case of Cuba that produced vaccines from the public sector in a record time (13). For example, the Biomanghinos laboratory and the Butantan institute in Brazil^13^,^14^ have expanded their contributions during the pandemic times, promoting the local development of a recombinant antigen for SARS-CoV-2. Also in India, Bharat Biotech has developed COVAXIN^®^, in collaboration with the Indian Council of Medical Research (ICMR)—National Institute of Virology (see text footnote 2). Recently, Argentina joined the group of the 10 countries in the world that locally produced a COVID-19 vaccine devised by the public sector and then finally developed through a public-private consortium (4).^15^ These successful initiatives make us wonder about the possibilities that other peripheral countries may have to promote their own research and developments. We believe that there are several approaches that countries as Argentina could implement by determining priorities and strategies in health research agendas, especially during sanitary emergencies.

Firstly, the COVID-19 pandemic has demonstrated that long-term planning of an integral scientific policy that promotes strategic topics is needed, as emergency health crisis preparedness was found to be insufficient in most countries.^16^ Therefore, from a lesson-learned perspective, peripheral countries should work on public initiatives that could help gaining infrastructure and accelerated developments on curative as well preventive implementations, especially in the case of infectious diseases, to gain independence from central countries capabilities. In the case of Argentina, all the knowledge and industrial development in the biotechnological sector gained thanks to public funding could hopefully be positively monopolized in a future emergency crisis to attend National and Regional demands.

Secondly, the articulation between the science and health ministries has a pivotal role to cope with eventual health emergencies. This would help to guide the research questions to the needs of the society. One initiative that was taken in Argentina in 2021 in this direction, was the inclusion of National Universities in the National Agency of Public Laboratories (ANLAP^17^). This interface allows interconnecting the development and production of pharmacological therapies focused on the local needs, involving agents from the public health till basic research areas. This helps not only in actual therapeutics developments, but also to promote active research in the field of preventive and palliative therapies for the local population. Therefore, these types of initiatives could also reinforce public strategies facing future sanitary emergencies.

Thirdly, interdisciplinary collaborations in networks and consortiums among researchers of different fields should be promoted to help with public health agenda issues. This would improve the achievements of solutions from different perspectives and also their quicker materialization. This was observed during the pandemic, in which researchers working in chemistry and physics of materials, developed masks with a particular nanotechnology that repelled the SARS-CoV-2 virus (Atom-protect^18^). However, as we previously mentioned, this type of collaborative research is not always taken into account by the scientific evaluation system nor by peer reviewer journals. This fact discourages young researchers to get involved in these types of initiatives due to the possible negative impact on their scientific career. Therefore, the scientific and financial support of interdisciplinary and collaborative networks locally and internationally should start to be considered for the development of public agendas, as well as positively regarded as a step in the scientific career, especially for young researchers. For example, from a consortium perspective, a good way to achieve this may be to ensure engaging researchers from different research fields and institutions to promote better representation of different contexts and expertise. To reinforce this strategy, scientists with different affiliations and from diverse fields might be considered as corresponding authors in the scientific papers produced by consortiums. From the scientific evaluation perspective, a practical guide with clear criteria for fair evaluation of this kind of initiatives should be designed in every National research council. Particularly, multidisciplinary committees should be summoned to deliver those practical guides and to better analyze the outcomes as well as the fair contributions of these collaborative initiatives. An example of a policy perspective of this kind of initiative are the Horizon Projects promoted by the EU^19^ where scientists from different fields and contexts are eligible to be financed to address a particular problem of humankind.

## 3 Discussion

This article aimed to discuss our personal experience and outcomes as members of the so-called AntiCovid Consortium during the COVID-19 pandemic in a peripheral country like Argentina. We presented the positive results and the challenges in terms of financial and scientific recognition that we faced during this period. After analyzing these insights, new aspects opened in terms of scientific agendas as well as the role of collaborative initiatives in the scientific system from the collective to the individual perspective. In order to contribute the analysis and future perspectives on this matter, we outlined some practical recommendations for establishing a stronger scientific system that can provide preventive and therapeutic tools developed by national and public systems, especially in sanitary emergencies. Here, we pointed out that collaborative initiatives have a scientific and social role in the community, and therefore they should be considered and promoted among the scientific system. We consider that the financial support of these kinds of initiatives could lead to fruitful outcomes not only for research projects but also for quicker transference both to the health and the productive sector. All of these considerations could help in promoting the connection between the scientific and public health agendas.

## Author contributions

NF: Conceptualization, Writing – original draft. MH: Conceptualization, Writing – original draft. MB: Writing – review & editing. MP: Conceptualization, Writing – original draft.

## Footnotes

^1^ https://portal.fiocruz.br/vacina-covid-19-vacinas-em-desenvolvimento

^2^ https://www.bharatbiotech.com/covaxin.html

^3^ https://anticovidarg.wixsite.com/consorcioanticovid

^4^ https://www.dw.com/en/coronavirus-argentinas-never-ending-quarantine/a-54721129

^5^ https://fundacen.org.ar/en/

^6^ https://www.argentina.gob.ar/ciencia/agencia

^7^ https://www.vetanco.com/en/2020/06/03/bioinnovo-inoculated-first-batch-birds-rbd-receptor-binding-domain-protein-sars-cov-2-develop-igy-possible-use-treatment-diagnosis/

^8^ https://www.argentina.gob.ar/sites/default/files/guia_para_ventilar_la_escuela.pdf

^9^ http://www.dii.uchile.cl/~ris/RIS2020/p2_iniciativas_uba_covid19_argentina.pdf

^10^ https://www.argentina.gob.ar/noticias/nuevos-financiamientos-para-el-diseno-de-vacunas-argentinas-contra-la-covid-19-0

^11^ https://www.conicet.gov.ar/un-kit-para-detectar-anticuerpos-de-sars-cov-2-desarrollado-por-cientificas-del-conicet-comenzara-a-producirse-en-un-laboratorio-publico-de-la-provincia-de-chaco/

^12^ https://www.fiercepharma.com/manufacturing/astrazeneca-to-supply-millions-covid-19-shot-to-brazilian-government-swamped-by-new

^13^ https://www.bio.fiocruz.br/index.php/en/

^14^ https://butantan.gov.br/

^15^ https://www.argentina.gob.ar/noticias/esta-lista-la-primera-vacuna-de-fabricacion-argentina-arvac-cecilia-grierson

^16^ https://www.oecd.org/coronavirus/policy-responses/first-lessons-from-government-evaluations-of-covid-19-responses-a-synthesis-483507d6/#textbox-d1e30

^17^ https://www.argentina.gob.ar/salud/anlap

^18^ https://www.conicet.gov.ar/scientists-developed-anti-viral-fabric-for-coronavirus-masks/

^19^ https://research-and-innovation.ec.europa.eu/system/files/2022-06/ec_rtd_he-investing-to-shape-our-future_0.pdf
